# Association of adiposity with thyroid nodules: a cross-sectional study of a healthy population in Beijing, China

**DOI:** 10.1186/s12902-019-0430-z

**Published:** 2019-10-16

**Authors:** Hui-xia Yang, Yu Zhong, Wei-hua Lv, Feng Zhang, Hong Yu

**Affiliations:** Beijing Rehabilitation Hospital, Capital Medical University, Xixiazhuang, Badachu, Shijingshan District, Beijing, 100144 China

**Keywords:** Adiposity, Thyroid nodules, Body mass index, Visceral fat area

## Abstract

**Background:**

The relationship between thyroid nodules (TNs) and adiposity is controversial. This paper describes a cross-sectional investigation performed to determine the existence of any such relationship. To assess adiposity, body mass index (BMI) and visceral fat area (VFA) were utilized.

**Methods:**

Between January 1, 2017 and March 3, 2019. Three thousand five hundred thirty four healthy people were examined using thyroid ultrasonography, visceral fat and anthropometric measurements, laboratory tests and questionnaire interview. Binary logistic regression analyses were used.

**Results:**

Of the 3534 healthy subjects, 58.69% (2074/3534) of the subjects had TNs. A total of 55.91% (1976/3534) had BMI ≥ 25 kg/m^2^ and 39.67% (1402/3534) had VFA ≥ 100 cm^2^. After adjustment to address confounders, BMI-based overweight and obesity levels only correlated with higher risk TNs when used as a continuous variable (OR = 1.031, 95% CI: 1.008–1.055, *P* = 0.008), while VFA was both a continuous variable (OR = 1.003, 95% CI: 1.000–1.005, *P* = 0.034) and a categorical variable (OR = 1.198, 95% CI: 1.014–1.417, *P* = 0.034) associated with significantly elevated risk of TNs. Analyzing the subgroups, BMI ≥ 25 kg/m^2^ (OR = 1.500, 95% CI: 1.110–2.026, *P* = 0.008) was significantly correlated with TN risk in individuals with TG ≥ 1.7 mmol/L. VFA ≥ 100 cm^2^ correlated with the TN risk irrespective of age (< 50 years: OR = 1.374, 95% CI: 1.109–1.703, *P* = 0.004; ≥ 50 years: OR = 1.367, 95% CI: 1.063–1.759, *P* = 0.015) and in the following subgroups: women (OR = 4.575, 95% CI: 2.558–8.181, *P* = 0.000), FBG ≥ 6.1 mmol/L (OR = 1.522, 95% CI: 1.048–2.209, *P* = 0.027), and TG ≥ 1.7 mmol/L (OR = 1.414, 95% CI: 1.088–1.838, *P* = 0.010).

**Conclusions:**

Adiposity correlates with TNs. To assess TN risk in Chinese individuals, VFA is better than BMI.

## Background

Among the most prevalent diseases in the population are thyroid nodules (TNs), the incidence of which is rising along in parallel with technological progress in treatments and diagnosis, lifestyle changes and environmental pollution. Many reports have stated that between 4 and 7% of TNs are diagnosed by palpation and 19 and 67% are found using high-resolution ultrasound [1]; between 8 and 16% of these TNs were diagnosed as cancer [2]. Delimitation of risk factors related to TNs is considered an important aim.

The association between TNs and adiposity have been investigated by many studies [1, 3–5]. The measurement of adiposity in these studies was primarily body mass index (BMI) and waist circumference. BMI is used to assess the severity of obesity; however, fat distribution, in particular the proportion between subcutaneous and visceral fat, is not determined by BMI [6]. Waist circumference reflects not only pre-peritoneal and visceral but also subcutaneous fat accumulation and is related to inter-ethnic variability and significant inter-operator differences [7–9]. Previous studies have reached controversial conclusions regarding the association among TN risks and adiposity; two Chinese studies in specific communities reported a higher risk of TNs in women associated with obesity and overweight (measured using BMI) [5, 10]. Another investigation did not find a relationship between TNs and BMI [11]. Of note, this was a study aimed at community-based population over 40 years of age in Nanjing, China. It is possible that the relationship between TNs and BMI may depend on different age groups and thyroid functions, which need further studies for clarification. Moreover, other important growth factors and complex interactions between genetic and environmental factors may also be involved in the etiology of TNs independently of BMI [12, 13].

Adipose tissue distribution, and particularly visceral fat area (VFA), can be accurately quantified using computed tomography, magnetic resonance imaging, and bioelectrical impedance analysis [14–16]. It is another index that evaluates adiposity that has been shown to correlate well with visceral fat mass [17]. We hypothesized that the VFA would be associated with TNs, and could be used as an indicator for TNs. However, no studies investigated the relationship between TNs and VFA until now.

We aimed to bring additional clarity to the association of TNs with adiposity, using BMI and VFA as the measurement of adiposity. BMI and VFA were compared to determine the better indicator of TNs risk.

## Methods

### Study participants

The study was carried out among healthy residents in Beijing, at Beijing Rehabilitation Hospital between January 1, 2017 and March 1, 2019. All subjects gave their informed consent for inclusion before they participated in the study. The study was conducted in accordance with the Declaration of Helsinki, and the protocol was approved by the Ethics Committee of Beijing Rehabilitation Hospital, Capital Medical University. The inclusion criteria included: (1) age over 25 years and under 90 years; (2) history of regular check up at the medical center in the past; and (3) meeting the requirements of the SENIEUR Protocol on the definition of healthy adults [18]. The exclusion criteria included: (1) lactation or pregnancy; (2) history of thyroid surgery; (3) cancer, renal insufficiency or adrenocortical insufficiency; and (4) treatment affecting iodine excretion or thyroid function, including amiodarone, antiepileptic drugs and glucocorticoids. According to the requirements of logistic regression analysis on sample size [19], the sample size should be at least 10 times that of the number of independent variables. In this study, a total of 12 independent variables were input in the logistic regression models; therefore, the sample size should be at least 12 × 10 = 120. According to a previous study, the incidence of TNs among the Chinese population was 12.62% [20]. Therefore, the number of participants should be at least 120÷12.62% ≈ 951.

### Data collection

After obtaining written informed consent, the eligible participants were asked to fast overnight and were interviewed by trained personnel using a structured questionnaire about thyroid disease and other conditions, smoking and high salt intake status and demographic characteristics. A similar questionnaire was used by Bin Song et al. [20]. Participants were also examined in terms of height (measured to the nearest 0.1 cm) and weight (measured to the nearest 0.1 kg), measured using an ultrasonic scale and height measuring tool (HNH-318, Omron, Japan) with the individuals wearing light clothes without shoes. Their diastolic blood pressure (DBP) and systolic blood pressure (SBP) (measured to nearest 1 mmHg) were measured using an electronic sphygmomanometer (HBP-9020, Omron, Japan) when the individuals were seated and quiet, taking two measurements with a separation of 30 s between each measurement, averaging the results. Their blood was taken and sent to the clinical laboratory of Beijing Rehabilitation Hospital for measurement of uric acid (UA), fasting blood glucose (FBG), triglycerides (TG), low density lipoprotein cholesterol (LDL-C), high density lipoprotein cholesterol (HDL-C) and total cholesterol (TC) levels. A color Doppler ultrasonic diagnosis tool (DC-7, Mindray, China) was used for thyroid ultrasonography. A visceral fat measurement device (HDS-2000, Omron, Japan) was used to measure VFA. All measurements were taken on a same day for each participant.

### Definition of variables

A TN was defined as a small lesion differentiated from the rest of the thyroid parenchyma, possessing a solid part, with or without the presence of a cystic part [21]. Ultrasonography characteristics of the TNs include the following: size, border, echogenicity, calcification and vascularity. Size was divided into ≥1 cm or < 1 cm. The boundaries were classified as ill-defined or well-defined (clear definition among surrounding parenchyma and the nodule). Echogenicity of the nodule was characterized as hypoechogenicity (hypoechoic in comparison with adjacent muscles or hypoechoic in comparison to normal parenchyma), and no hypoechogenicity. Another classification was calcification (taking both coarse calcifications and microcalcifications into account) and no calcification. Vascularity was subdivided into central vascularity (noting color Doppler flow inside the nodule) and no central vascularity in this study. VFA was considered elevated if ≥100 cm^2^, following the adult obesity criteria established by the Japan Society for the Study of Obesity [22]. BMI was calculated as weight (kg) divided by height (m) squared, defining overweight as BMI ≥ 25 kg/m^2^ and obesity as BMI ≥ 30 kg/m^2^, following the World Health Organization criteria [23]. Normal reference ranges were as follows: FBG 3.9–6.1 mmol/L; TG 0.5–1.7 mmol/L; LDL-C 2.1–3.1 mmol/L; HDL-C 0.9–1.8 mmol/L; and TC 3.0–5.7 mmol/L. The FBG was considered high if ≥6.1 mmol/L, while elevated TG was defined as ≥1.7 mmol/L, following the World Health Organization guidelines. High salt intake was defined as eating more than 10 g of salt every day, including the amount of salt ingested through various condiments such as soy sauce, pickles and monosodium glutamate.

### Statistical analysis

Data analysis was performed using the Statistical Package for the Social Sciences, version 22 (SPSS Inc., Illinois, USA). Percentage and counts were used to report categorical data. Continuous data were described using mean and standard deviation (SD), or median and interquartile range (IR) if the data did not follow a normal distribution. The Kolmogorov-Smirnov test was used to determine whether the data were normally distributed. The chi-square test and unpaired t-test were used to assess the significance of the proportions or mean differences among individuals with or without TNs, respectively. Odd ratios (ORs) and 95% confidence intervals (95% CIs) were calculated using binary logistic regression analyses, in order to determine associations of TNs with BMI and VFA. Twelve probable risk factors, that is, TC, HDL-C, LDL-C, TG, FBG, UA, DBP, SDP, high salt intake and smoking status, age and gender were considered using adjusted logistic regression models. Subgroups analyses were performed using VFA and BMI as categorical variables and dividing the participants by four probable risk factors: gender, age (< 50 or ≥ 50 years), FBG (< 6.1 or ≥ 6.1 mmol/L), and TG (< 1.7 or ≥ 1.7 mmol/L). *P* < 0.05 was regarded as statistically significant. Forest plots were drawn using R software, version 3.6.0 (R Foundation for Statistical Computing, Vienna, Austria).

## Results

### Baseline characteristics

**Fig. 1:**
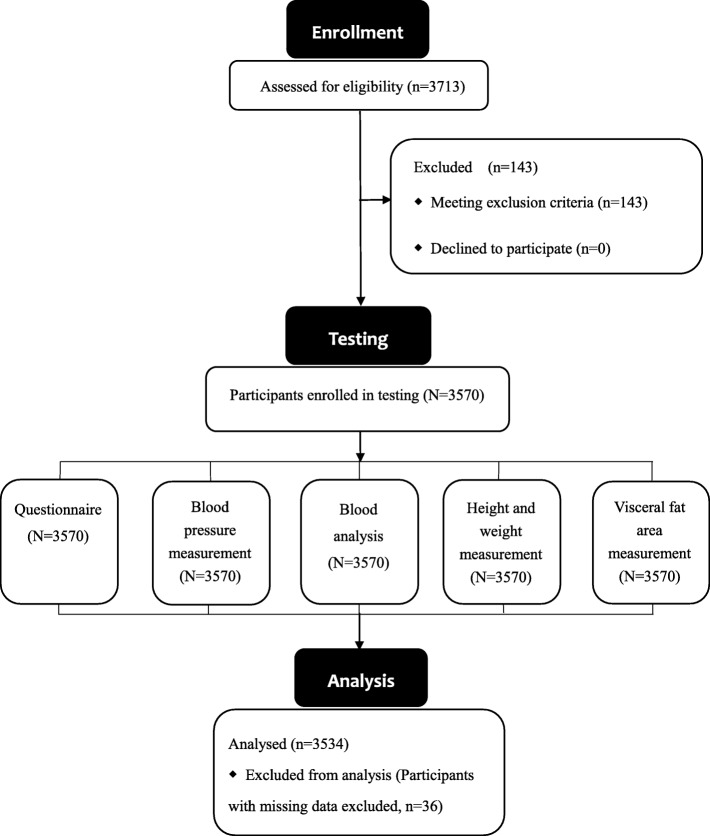
Flow diagram of screened, enrolled and tested participants

**Fig. 2:**
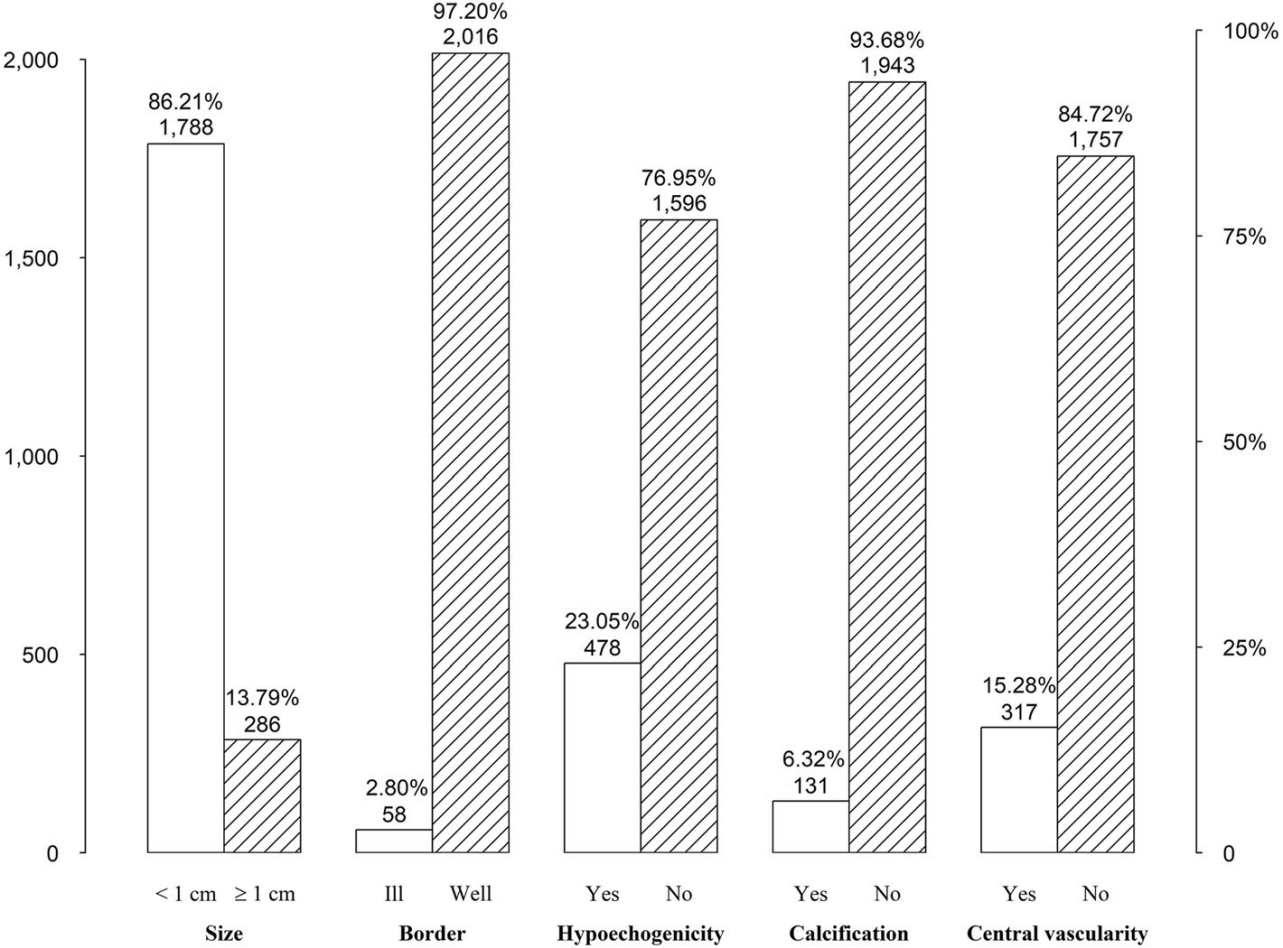
Ultrasonography characteristics of the thyroid nodules

After the exclusion of 36 subjects with missing data (14 subjects had missing questionnaire results, 12 subjects with missing VFA results, ten subjects with missing blood analysis results) and 143 subjects with previous thyroid diseases, 3534 healthy subjects (median age: 48.00 years, IR: 42.00–54.00 years) were included in the analysis (Fig. 1). Of the 3534 healthy subjects, 67.54% (2387/3534) were males and 54.88% (1310/2387) had TNs; 32.46% (1147/3534) were females and 66.61% (764/1147) had TNs. For the 2074 subjects with TNs, the size of the TNs ranged from 0.10 cm to 6.50 cm (median size: 0.45 cm, IR: 0.30–0.70 cm). The prevalence of TNs in females was significantly higher than that of males (*P* = 0.000). Of the 3534 healthy subjects, mean BMI was 25.78 ± 3.75 kg/m^2^ and mean VFA was 91.27 ± 39.42 cm^2^; 55.91% (1976/3534) of these subjects had BMI ≥ 25 kg/m^2^, and 39.67% (1402/3534) had VFA ≥ 100 cm^2^. The individuals with TNs were significantly older than those without TNs (50.54 vs. 45.97 years, *P* = 0.000) and had a significantly higher proportion of females (36.84% vs. 26.23%, *P* = 0.000), higher BMI (25.89 vs. 25.63 kg/m^2^, *P* = 0.036), higher VFA (92.74 vs. 89.19 cm, *P* = 0.008), higher SBP (129.83 vs. 127.45 mmHg, *P* = 0.000), and higher proportion of VFA ≥ 100 cm^2^ (61.34% vs. 38.66%, *P* = 0.009). Individuals with or without TNs were similar in terms of DBP, UA, TG, LDL-C, HDL-C, TC, smoking status, and high salt intake status (Table 1). The ultrasonography characteristics of the TNs are shown in Fig. 2.

**Table 1:** Baseline characteristics of the participants with or without thyroid nodules

Characteristic	No nodules (*N* = 1460)	Nodules (*N* = 2074)	*P*
Male	1077 (45.12%)	1310 (54.88%)	0.000
Female	383 (33.39%)	764 (66.61%)	
Age (years)	45.97 ± 8.43	50.54 ± 9.95	0.000
BMI (kg/m^2^)	25.63 ± 3.70	25.89 ± 3.78	0.036
VFA (cm^2^)	89.19 ± 39.29	92.74 ± 39.46	0.008
SBP (mmHg)	127.45 ± 17.28	129.83 ± 16.94	0.000
DBP (mmHg)	78.55 ± 12.39	79.02 ± 11.88	0.259
UA (μmol/L)	343.60 ± 88.01	344.26 ± 85.75	0.821
FBG (mmol/L)	5.58 ± 1.55	5.69 ± 1.74	0.038
TG (mmol/L)	1.69 ± 1.40	1.75 ± 1.33	0.207
LDL-C (mmol/L)	3.04 ± 0.80	3.07 ± 0.80	0.272
HDL-C (mmol/L)	1.30 ± 0.27	1.31 ± 0.28	0.111
TC (mmol/L)	4.88 ± 0.94	4.91 ± 0.94	0.386
Smoking status			
No	1005 (40.75%)	1461 (59.25%)	0.305
Currently	455 (42.60%)	613 (57.40%)	
High salt intake status			
No	1451 (41.29%)	2063 (58.71%)	0.737
Currently	9 (45.00%)	11 (55.00%)	
BMI ≥ 25 kg/m^2^	800 (40.49%)	1176 (59.51%)	0.261
VFA ≥ 100 cm^2^	542 (38.66%)	860 (61.34%)	0.009

Abbreviations: *N* number, *BMI* body mass index, *VFA* visceral fat area, *SBP* systolic blood pressure, *DBP* diastolic blood pressure, *UA* uric acid, *FBG* fasting blood glucose, *TG* triglycerides, *LDL-C* low density lipoprotein cholesterol, *HDL-C* high density lipoprotein cholesterol, *TC* total cholesterol

### Associations of TNs with BMI and VFA

**Fig. 3:**
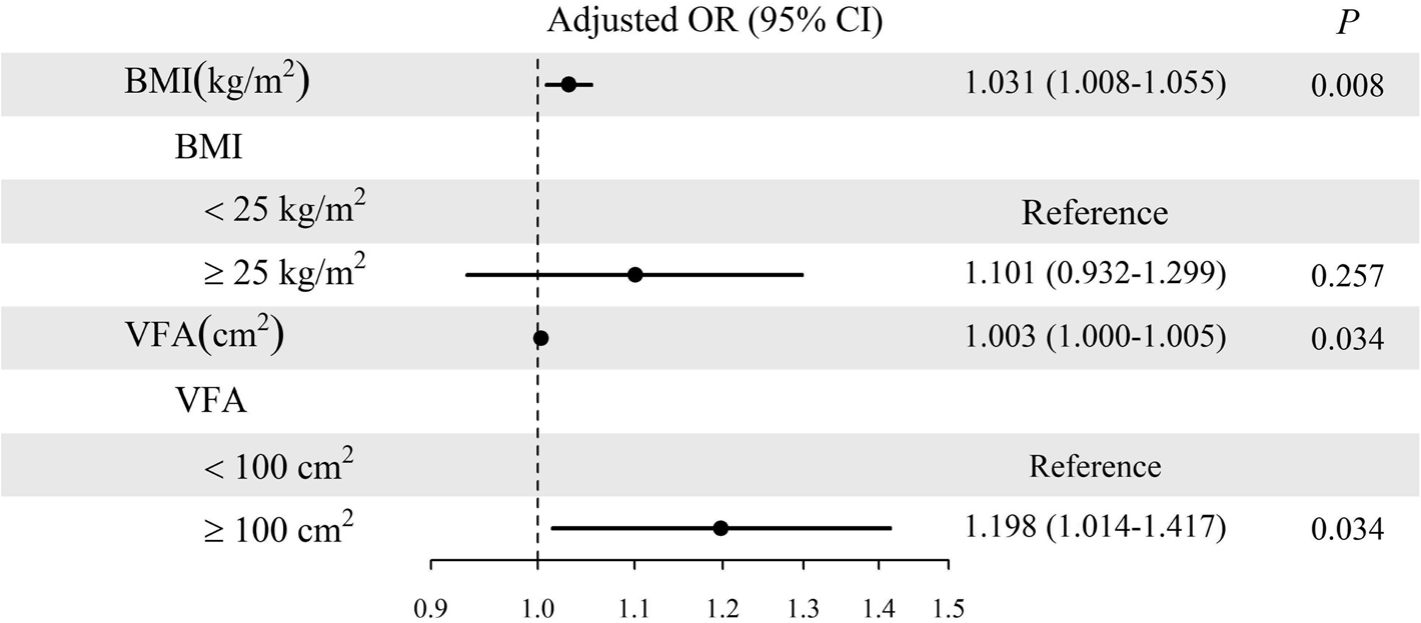
Analysis of associations of thyroid nodules with BMI and VFA. Abbreviations: OR, odd ratio. The adjusted OR controls for gender, age, smoking and high salt intake status, SBP, DBP, UA, FBG, TG, LDL-C, HDL-C and TC

Figure 3 shows the relationships among TNs and VFA or BMI over the whole study sample. The continuous variable BMI was associated with a higher risk of TNs in the unadjusted model (OR = 1.019, 95% CI: 1.001–1.038, *P* = 0.037), maintaining its statistical association with TNs after adjusting the model for gender, age, smoking, high salt intake status, SBP, DBP, UA, FBG, TG, LDL-C, HDL-C and TC (OR = 1.031, 95% CI: 1.008–1.055, *P* = 0.008). The categorical variable BMI was not significantly associated with TNs in either the unadjusted or the adjusted model: the OR for individuals with BMI ≥ 25 kg/m^2^ relative to those with BMI < 25 kg/m^2^ was 1.080 (95% CI: 0.944–1.236, *P* = 0.261) in the unadjusted model and the OR in adjusted model was 1.101 (95% CI: 0.932–1.299, *P* = 0.257). The continuous variable VFA was significantly related to higher risk of TNs in the unadjusted model (OR = 1.002, 95% CI: 1.001–1.004, *P* = 0.008) and also in the adjusted model (OR = 1.003, 95% CI: 1.000–1.005, *P* = 0.034). The categorical variable VFA maintained its significant relation with TNs in both unadjusted (OR = 1.272, 95% CI: 1.076–1.504, *P* = 0.005) and adjusted (OR = 1.198, 95% CI: 1.014–1.417, *P* = 0.034) models.

### Subgroup analyses on gender, age, FBG and TG

**Fig. 4:**
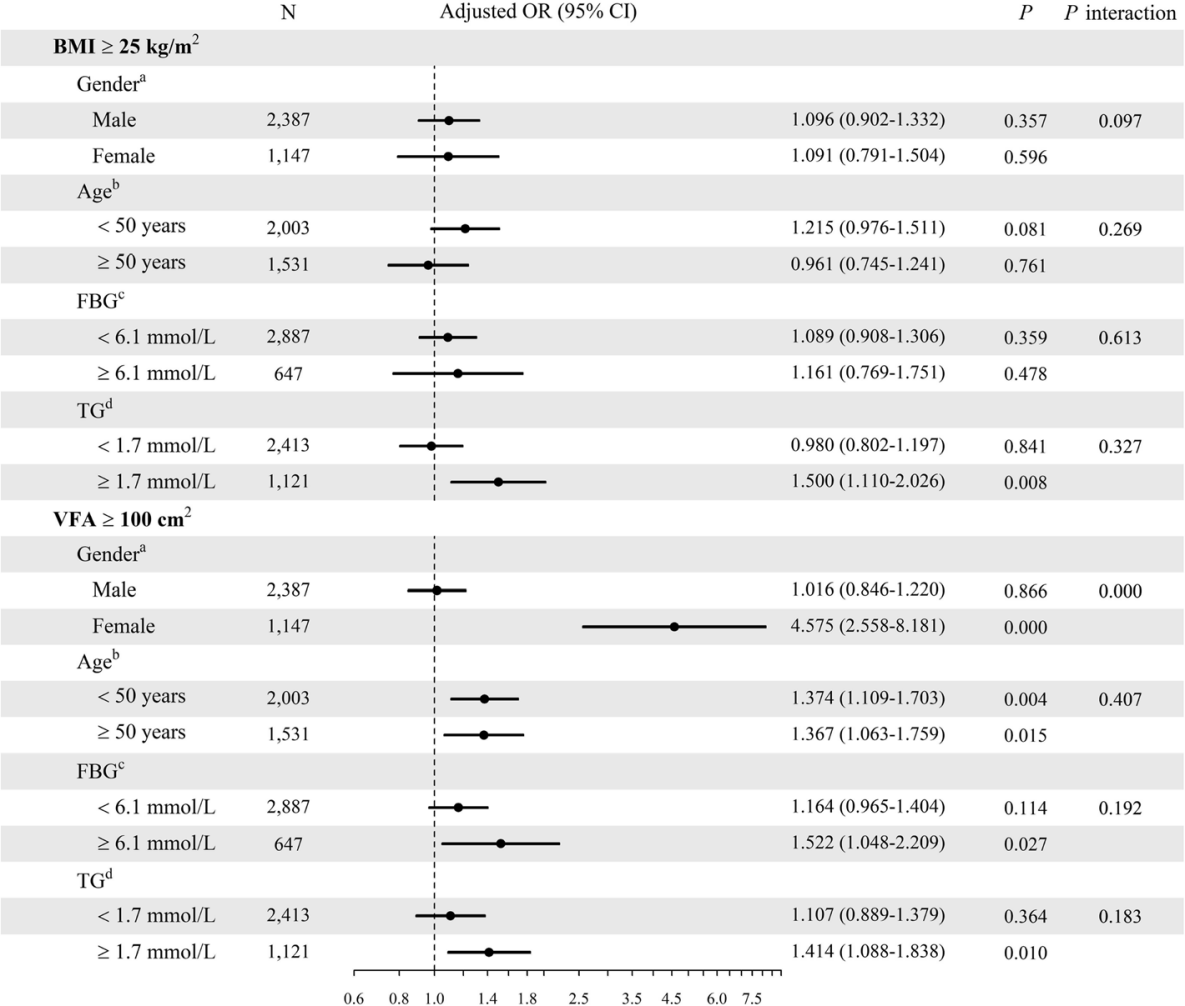
Associations of thyroid nodules with BMI and VFA in subgroups of subjects stratified by gender, age, FBG or TG. ^a^Adjusted for age, smoking and high salt intake status, SBP, DBP, UA, FBG, TG, LDL-C, HDL-C, TC. ^b^Adjusted for gender, smoking and high salt intake status, SBP, DBP, UA, FBG, TG and LDL-C, HDL-C, TC. ^c^Adjusted for gender, age, smoking and high salt intake status, SBP, DBP, UA, TG and LDL-C, HDL-C, TC. ^d^Adjusted for gender, age, smoking and high salt intake status, SBP, DBP, UA and LDL-C, HDL-C, TC

Potential relations of TNs with VFA or BMI, both considered as categorical variables, were explored in various subgroups of study participants (Fig. 4). BMI ≥ 25 kg/m^2^ was associated with higher TN risk significantly only in individuals with TG ≥ 1.7 mmol/L (OR = 1.500, 95% CI: 1.110–2.026, *P* = 0.008), while VFA ≥ 100 cm^2^ significantly correlated with increased risk of TNs in women (OR = 4.575, 95% CI: 2.558–8.181, *P* = 0.000), individuals < 50 years old (OR = 1.374, 95% CI: 1.109–1.703, *P* = 0.004), individuals ≥50 years old (OR = 1.367, 95% CI: 1.063–1.759, *P* = 0.015), individuals with FBG ≥ 6.1 mmol/L (OR = 1.522, 95% CI: 1.048–2.209, *P* = 0.027) and individuals with TG ≥ 1.7 mmol/L (OR = 1.414, 95% CI: 1.088–1.838, *P* = 0.010). Only VFA ≥ 100 cm^2^ (*P* for interaction = 0.000) had a statistically significant interaction effect with gender.

## Discussion

This study suggests that adiposity correlates with TNs. It is possible that adiposity was associated with insulin resistance and increased production of insulin and insulin-like growth factors, which in turn was associated with thyroid disorders [24–26]. Moreover, a investigation demonstrated the relationship among thyroid gland morphologic alterations and adiposity, as an excess of it can cause thyroid steatosis, which is an expansion in the adipose depot between follicular cells in the thyroid [27].

In the present study, elevated VFA is more strongly associated with higher risk of TNs than it is associated with BMI. BMI was associated with higher TNs risk only as a continuous variable, but not when defined as a categorical variable. This is similar to findings from previous studies. Bin Song et al. reported that waist circumference was superior to BMI for assessing risk of TNs in Chinese subjects. Weimin Xu et al. found there is a stronger relationship among TNs and body surface area than among TNs and BMI, both of which can be used to better indicate the risk of TNs in presence of obesity and overweight. This lack of accuracy in the predictability or as an indicator of BMI for TNs risk may be due to the fact that it is a nonspecific measurement method for adiposity, merging bone and abdominal adipose tissue and muscle mass measurements [28].

VFA was associated with higher TN risk both as a categorical and as a continuous variable, suggesting a more important role of VFA in the predictability of TNs risk. Many researchers have found that accumulation of visceral fat can promote lipid synthesis because of the presence of increased amounts of free fatty acids, inducing metabolic syndrome through glycogen heterogenesis [29]. The main feature of the metabolic syndrome, insulin resistance, may cause hyperplasia of thyroid cells, inducing TNs and thyroid cancer [4].

Subgroup analysis demonstrated that VFA ≥ 100 cm^2^ was significantly related to TN risk in almost all groups, while BMI ≥ 25 kg/m^2^ was only significantly related to increased risk of TN in the TG ≥ 1.7 mmol/L group. This supports the notion that VFA ≥ 100 cm^2^ is more accurate for indicating TN risk than a general obesity measurement such as BMI.

We found that the prevalence of TNs in women was higher than that of men. Subgroup analysis also resulted in the finding of a higher risk of TNs in women with VFA ≥ 100 cm^2^ than in men with VFA ≥ 100 cm^2^. There have been many studies reporting the association between TNs and women. Ju-Yeon found that healthy Korean women who were < 160 cm and ≥ 60 kg were at risk of TNs [30]. Xu found that height, body mass, BMI and body surface area were particularly associated with the risk of TNs among Chinses women [10]. Chen also found that, in women, obesity, central obesity, and nonalcoholic fatty liver disease might contribute to taller-than-wide nodule development [31]. However, causes for these phenomena are unclear. The greater prevalence of thyroid nodular disease in female population may be caused by progesterone and estrogen. Kung et al. reported that pregnancy was associated with increase in size of preexisting TNs and new TN formation [32]. Another study reported that serum TSH was slightly higher in users of oral contraceptives [33]. The exact role of the gender effect on the thyroid needs to be further explored. Nevertheless, for women, especially those with visceral fat obesity, physicians should pay attention to thyroid health.

The current study has some limitations: 1) it was a cross-sectional study and did not reflect any temporal impact; 2) the numbers of subjects in our study were modest; 3) we did not test thyroid function and therefore could not take them as cofounders when analyzing the data; and 4) this was a single-center study; therefore, the results may not be generalizable. Nevertheless, this study is the first report for the relationship between VFA and TNs; we set strict inclusion and exclusion criteria, our study subjects were healthy individuals without thyroid surgery or treatment that may affect the function of thyroid and the excretion of iodine, and we used binary logistic regression analysis to control confounding factors when analyzing the association of TNs with BMI and VFA. Therefore, the present findings remain valuable. In the future, a multicenter study with a larger sample size, a more detailed collection of biochemical indicators, a comprehensive questionnaire, and a long follow-up period may provide more valuable data.

## Conclusions

Adiposity is related with TNs. Elevated VFA is more strongly associated with higher risk of TNs than it is associated with BMI, possibly serving as a potential indicator for TNs risk. Maintaining normal VFA may be helpful to avoid the appearance of TNs, especially in women.

## Data Availability

The datasets used and/or analysed during the current study are available from the corresponding author on reasonable request.

## Abbreviations

BMI: Body mass index
CIs: Confidence intervals
DBP: Diastolic blood pressure
FBG: Fasting blood glucose
HDL-C: High density lipoprotein cholesterol
LDL-C: Low density lipoprotein cholesterol
ORs: Odd ratios
SBP: Systolic blood pressure
SD: Standard deviation
TC: Total cholesterol
TG: Triglycerides
TNs: Thyroid nodules
UA: Uric acid
VFA: Visceral fat area
